# Natural killer cell levels in adults living with type 2 diabetes: a systematic review and meta-analysis of clinical studies

**DOI:** 10.1186/s12865-020-00378-5

**Published:** 2020-09-09

**Authors:** Vuyolwethu Mxinwa, Phiwayinkosi V. Dludla, Tawanda M. Nyambuya, Kabelo Mokgalaboni, Sithandiwe E. Mazibuko-Mbeje, Bongani B. Nkambule

**Affiliations:** 1 School of Laboratory Medicine and Medical Sciences (SLMMS), College of Health Sciences, University of KwaZulu-Natal, Private Bag X54001, Durban, 4000 South Africa; 2 Department of Life and Environmental Sciences, Polytechnic University of Marche, Ancona, Italy; 3 Biomedical Research and Innovation Platform, Medical Research Council, Tygerberg, South Africa; 4 Department of Health Sciences, Faculty of Health and Applied Sciences, Namibia University of Science and Technology, Windhoek, 9000 Namibia; 5 Department of Biochemistry, Faculty of Natural and Agricultural Sciences, North-West University, Mmabatho, South Africa

**Keywords:** Cardiovascular diseases, Inflammation, Natural killer cells, Type 2 diabetes mellitus

## Abstract

**Background:**

Chronic immune activation and hyperglycaemia are a hallmark of type 2 diabetes mellitus (T2D) while natural killer (NK) cells are involved in the pathogenesis of T2D. Dysregulated NK cell responses are associated with an increased risk of cardiovascular disease in patients living with T2D.

**Objective:**

To provide a comprehensive and systematic evidence-based estimate on the levels of NK cells in patients living with T2D.

**Results:**

This systematic review and meta-analysis included 13 studies reporting on 491 adult patients with T2D and 1064 nondiabetic controls. The pooled effect estimates showed increased levels of NK cells in adult patients with T2D compared to controls (MD: 0.03 [− 3.20, 3.26], I^2^ = 97%, *p* < 0.00001).

**Conclusion:**

Overall, the evidence presented in this systematic review shows that the changes in NK cells in patients living with T2D are still unclear and further studies are needed.

## Background

Non-communicable disease such as type 2 diabetes (T2D) are amongst the leading causes of mortality and continue to burden the healthcare system of both developing and developed countries [1]. Globally, more than 425 million adults are living with diabetes, with T2D accounting for approximately 90% of the cases [2]. In developing countries such as those in sub-Saharan Africa, the prevalence of T2D is expected to increase by more than two-fold in the next 30 years and this coincides with an increased risk of cardiovascular disease (CVD) [3]. Obesity has been identified as a major risk factor for T2D [4].

Notably, over 90% of patients with T2D are obese [5] and these individuals present with ectopic lipid accumulation that is associated with adipose tissue (AT) dysfunction [6]. Although mechanisms involved in AT dysfunction in T2D are complex, enhanced infiltration of leukocytes into the AT promotes low-grade chronic inflammation which is mediated by T-helper (Th) 1 and Th17 responses [7]. Interestingly, our group and others have provided cumulative findings showing that persistent Th1 and Th17 cytokine levels which include tumor necrosis factor-α (TNFα), interleukin-1 (IL-1), IL-6 and IL-17 exacerbates insulin resistance which may ultimately lead to cardiovascular complications [8–12]. Furthermore, natural killer (NK) cells are constituents of the AT resident immune cell population which orchestrate a localised inflammatory response [12, 13]. A dysregulation of these immune cells promotes insulin resistance [13, 14]. There has been a growing interest in the role of NK in T2D, and in the development of T2D associated complications. In fact, a direct association between elevated NK cell levels and an increased risk of CVD has been reported [15]. Contradictory findings on the role of NK cells in exacerbating inflammation and insulin resistance in T2D exist [16, 17]. Thus, the role of NK cells in modulating T2D and its associated complications remains elusive.

There is currently no meta-analysis available aimed at providing an estimate of the levels of NK cells in patients living with T2D, who may also at risk of developing CVD. Therefore, this systemic review and meta-analysis aimed at providing a comprehensive synthesis of available literature elucidating the role of NK cell function in T2D. Since cardiovascular complications remain amongst the leading causes of death in patients with T2D [17] it is crucial to establish the relationship between NK cells and the development of T2D.

## Results

### Study selection

**Fig. 1:**
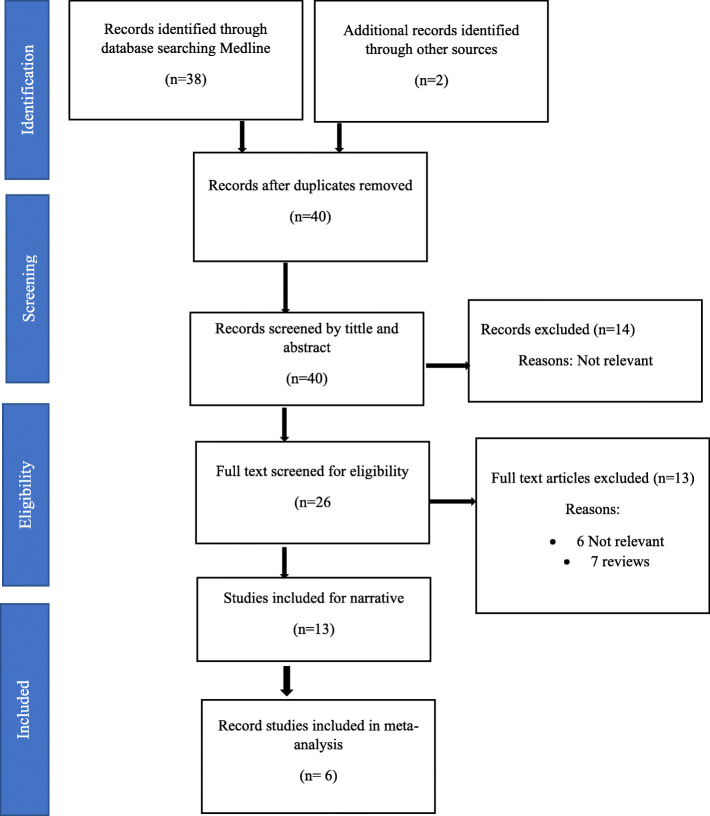
PRISMA flow diagram illustrating the study selection procedure

We identified a total of 40 citations (38 MEDLINE and 2 from other sources). A total of 14 citations were excluded after screening the title and the abstracts (Fig. 1). The remaining 26 studies were assessed for full-text eligibility. In all, 7 reviews and 6 studies were excluded as these were irrelevant (Fig. 1). We reported the findings of this systematic review and meta-analysis following the Meta-analysis Of Observational studies in epidemiology (MOOSE) guidelines [18]. In total, 13 studies fulfilled the study selection criteria and were included in the qualitative analysis [14–22, 24, 25, 27, 28, 35]. While only six studies were included in the meta-analysis [14, 15, 19–21, 35].

### Characteristics of included studies

The characteristics of the included studies are provided in Table 1. The 13 included studies were published in between 2008 and 2019. Additionally, the geographic distribution of the included studies comprised of Europe (*n* = 6) [14–17, 21, 27], Northern America (*n* = 2) [20, 23], Africa (*n* = 1) [24], and Asia (*n* = 4) [19, 25, 27, 28]. Two studies were longitudinal prospective studies [22] and the remaining 9 studies were cross-sectional studies [15–17, 21, 23, 26, 27, 29–31]. In total, this systematic review included 1555 adult participants (491 patients with T2D and 1064 controls) (Table 1). The age of the enrolled participants ranged from 16 to 70 years and comprised of 51% males.

**Table 1:** Characteristics of included studies (*n* = 13)

Author	Country	Study design	Sample size	Age	Male n (%)	Outcomes
Akesson, 2010 [15]	Sweden	Cross- sectional	46 T2D 20 Controls	T2D (55.8 ± 43.4) Control (NR)	38 (83)	Patients with T2D had decreased peripheral natural killer (NK) cells compared to controls.
Berrou, 2013 [16]	France	Cross- sectional	51 T2D 54 Controls	T2D (51.3 ± 14.4) Control (NR)	33 (65)	The levels of NK cells were similar between patients with T2D and healthy controls.
Guo, 2012 [27]	China	Cross- sectional	16 T2D 9 Controls	T2D (40 ± 35.7) Control (45 ± 20.4)	9 (56)	There was no significant difference in the levels of NK cells between T2D patients and control.
Kim, 2019 [28]	S. Korea	Cross- sectional	21 T2D, 13 Controls	T2D (60.71 ± 6.99) Control (55 ± 13.76)	8 (38)	Patients with diabetes had lower NK cell activity compared with participants with controls. Comparable levels of lipid profiles was reported between T2D and controls.
Lynch, 2009 [35]	Ireland	Cross- sectional	26 T2D 26 Controls	T2D (45.5 ± 5.8) Control (42.6 ± 9.8)	7 (27)	The levels of circulating NK cells were decreased in T2D compared to controls. NK cells from T2D, expressed elevated levels of the inhibitory markers NKB1 and CD158b as well as CD69, an activation marker. Comparable levels of high-density lipoprotein (HDL) and low-density lipoprotein (LDL) were observed between T2D patient and control. However, triglycerides levels were higher in T2D group in comparison to controls.
Medellin-Garibay, 2015 [23]	Mexico	Prospective cohort	18 T2D 24 Control	T2D (45.4 ± 11.4) Control (35.8 ± 6.6)	9 (50)	The levels of NK cells in circulation were similar between T2D and the control. Patients with T2D had increased levels of triglycerides, total cholesterol and LDL coupled with decreased Levels of HDL when compared to controls.
Simar, 2014 [21]	Denmark	Cross- sectional	6 T2D, 4 Controls	T2D (44.1 ± 6.5) Control (34.8 ± 3)	NR	There were no significant differences observed in the levels of NK cells between T2D groups and controls.
Nam, 2018 [25]	R. Korea	Cross- sectional	33 T2D 35 Controls	T2D (61.4 ± 5.9) Control (57.8 ± 5.3)	21 (64)	The level of NK cells and their functional activity were not different between T2D and control groups.
Nekoua, 2016 [24]	Benin	Cross- sectional	45 T2D 43 Controls	NR	21 (47)	The levels of NK cells were comparable between the T2D patients and the controls. Patients with T2D had lower levels of total cholesterol and triglycerides. However, there was no significant difference in LDL levels.
Olson, 2015 [20]	USA	Cross- sectional	154 T2D 775 Controls	T2D (66.9 ± 9.5) Control (65.4 ± 10)	84 (55)	No significant differences between the levels of NK cells in patients with T2D versus controls.
Piatkiewicz, 2013 [14]	Poland	Cross- sectional	18 T2D 18 Controls	T2D (53.6 ± 7.0) Control (52.4 ± 7.0)	9 (50)	Patients with T2D had significantly increased levels of NK cells in comparison to the healthy controls. However, the cytotoxic activity of NK cells in T2D individuals was reduced compared to controls.
Singh, 2019 [22]	Sweden	Cross- sectional	16 T2D, 13 Controls	T2D (64.0 ± 2.0) Control (61.6 ± 2.9)	NR (70)	Patients with T2D and healthy controls had comparable levels of NK cells
Xiaohong, 2018 [19]	China	Cross- sectional	41 T2D 30 Controls	NR	NR	Patients with T2D had significantly increased levels of peripheral NK cells compared to controls.

Not reported: NR; T2D: type 2 diabetes; NKB1: natural killer cell inhibitory receptor

### Risk of bias and quality of the studies

The risk of bias and quality of included studies was independently assessed by VM and TMN, using the modified Downs and Black’s checklist [37]. The overall scores were graded as, excellent (24–27); good (19–23); fair (13–18) and poor (≤12). Overall, the included studies were rated as poor, with an average score of 13 out of possible 26. Overall, the studies were scored as moderate for selection bias domain (with a score 7 out of possible 10), poor for internal validity (with a score 1 out of possible 3), moderate for external validity domain (scoring 3 out of possible 7), and moderate for reporting bias (with a score 3 out of possible 6). The inter-rater reliability per domain was scored as; k = 0.8 (CI = 0.62, 0.98) for selection bias (perfect agreement), k = 0.53 (CI = 0.3, 0.76) for internal validity (fair agreement), k = 0.43 (CI = 0.29, 0.56) for external validity (moderate agreement), and k = 0.67 (CI = 0.51, 0, 83) reporting bias (substantial agreement)*.*

### Primary outcomes

#### Levels of natural killer cells in patients living with T2D

**Fig. 2:**
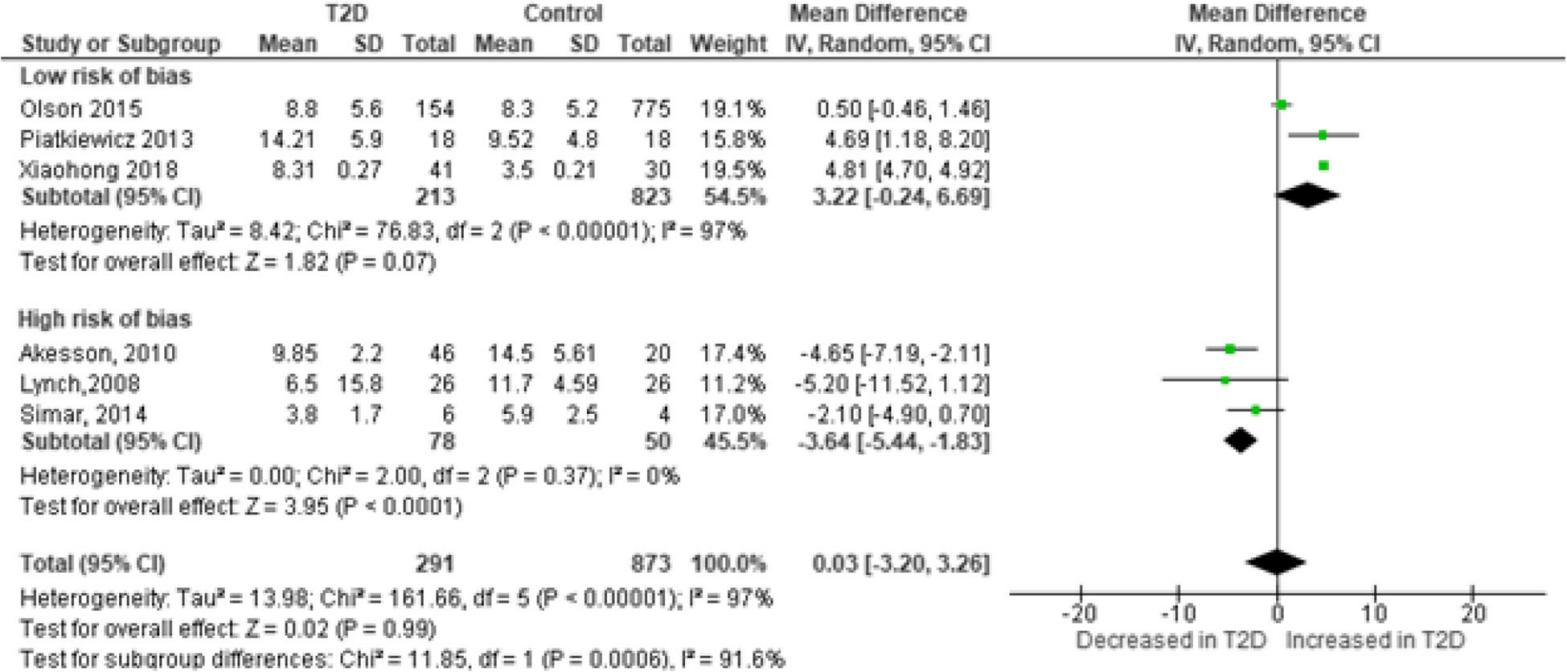
Levels of natural killer cells in patients living with T2D compared to controls

A total of six studies [16, 20, 22–25] reported no difference in the frequencies of circulating NK cells between patients with T2D and controls. Whilst others reported contradictory findings of decreased [25, 35] and increased [14] NK cells levels in patients with T2D. Notably, NK cells in patients with T2D expressed higher levels of the early activation marker CD69 [35] despite the reported lower frequency in T2D [15, 35]. The pooled estimates which included 6 studies reporting on 1165 participants showed a small effect size in the levels of NK cells betwenT2D patients and controls (MD: 0.03 [− 3.20, 3.26], I^2^ = 97%, *p* < 0.00001) (Fig. 2). To investigate the potential sources of substantial levels of statistical heterogeneity, we conducted a subgroup analysis based on the risk of bias. Notably, studies that were considered fair (≥13 points) showed an exacerbated increase in the levels of NK cells in T2D patients albeit high level of statistical heterogeneity (MD: 3.22 [− 0.24, 6.69], I^2^ = 97%, *p* < 0.00001). In contrast, studies that scored poor (≤12) showed a decreased levels of NK cells in T2D when compared to controls (MD: − 3.64 [− 5.44, − 1.83], I^2^ = 0%).

To further ascertain the robustness of our reported effect estimate of elevated levels on NK cells in T2D, we performed a sensitivity analysis based on participants’ smoking status which was previously reported to influence the levels of NK cells [29]. As a result, we omitted a study by Olson and colleagues which included T2D patients that were smokers and our results remained consistent and the level of statistical heterogeneity reduced to 0% (MD: 4.81 [4.70, 4.92]; I^2^ = 0%) (Table 2S). Notably, the potential sources of bias in the included studies were mainly due to differences in the external validity of the studies (Table 3S). In contrast, the selection and reporting bias ratings were comparable between the included studies (Table 3S). In the external validity sub-scale, studies that had a low risk of bias followed procedures to ensure that the included participants were representative of the population [14, 19, 20]. Whereas none of the studies that were rated as high risk reported on steps performed to validate the representativeness of the source population [15, 21, 35]. These studies obtained an overall score of 0 for the external validity domain (Table 3S) (Table 2).

**Table 2:** Summary of findings table

**T2D compared to control group**						
**Patient or population**: Adult individuals (> 18 years) **Exposure**: T2D **Comparison**: Non-diabetic (control) **Outcome**: Levels of peripheral blood natural killer cells						
Outcomes	Anticipated absolute effects^*^ (95% CI)		Relative effect (95% CI)	№ of participants (studies)	Certainty of the evidence (GRADE)	Comments
	**Risk with Control**	**Risk with T2D**				
Levels of peripheral blood natural killer cells		The mean levels in the exposure group was 0.03 higher (−3.20, 3.26)	–	1164 (6 observational studies)	⨁⨁oo LOW	
*The risk in the intervention group (and its 95% confidence interval) is based on the assumed risk in the comparison group and the **relative effect** of the intervention (and its 95% CI). **CI:** Confidence interval; **MD:** Mean difference; **OR:** Odds ratio; **NE**: Not estimable						
**GRADE Working Group grades of evidence** **High certainty:** We are very confident that the true effect lies close to that of the estimate of the effect**Moderate certainty:** We are moderately confident in the effect estimate: The true effect is likely to be close to the estimate of the effect, but there is a possibility that it is substantially different **Low certainty:** Our confidence in the effect estimate is limited: The true effect may be substantially different from the estimate of the effect**Very low certainty:** We have very little confidence in the effect estimate: The true effect is likely to be substantially different from the estimate of effect						

## Discussion

Delineating the pathological mechanisms involved in the development of T2D and its related complications remains crucial in the discovery of novel biomarkers of early disease, including establishing effective therapeutic strategies. There has been a growing interest in understanding the role of immunological mechanisms in the prognosis of inflammatory conditions. NK are the major innate lymphocyte subsets that are involved in the regulation of inflammatory response process and possess promising clinical exploration [30, 31]. The aim of this systemic review and meta-analysis was to provide a comprehensive synthesis of available studies reporting on NK cells in adult patients living with T2D.

The cumulative evidence presented in this study shows that the levels of NK cells are slightly elevated in patients with T2D [15, 21, 36]. However, there seems to be distinct difference in the regulation of NK cells in T2D [16, 17]. In particular, some studies reported on the comparable levels of NK cells between patients with T2D and nondiabetic controls [15, 16, 20, 23, 24]. However, peripheral blood NK cells expressed higher levels of CD69, an early activation marker that is essential for the modulation of inflammatory response [35]. In addition to CD69, NKG2D is another surface receptor, mainly identified in NK cells that is elevated in patients with T2D patients [15], although others reported lower levels of NKG2D in patients with T2D [16, 19]. The regulation of NKG2D in patients with T2D remains unclear. However, the stimulation of NK cells through NKG2D or NKp46, its activation receptor, may exacerbate the production of inflammatory cytokines [16]. Moreover, studies have reported on elevated levels of the inhibitory markers NKB1, CD158b, and KIR3DL-1 in patients with T2D when compared to controls [15, 19, 25, 26, 35].

These are inhibitory markers on the surface NK cells that regulate cytotoxic activity these cells. Furthermore, an imbalance between activation and inhibitory receptors expressed on NK cells may compromise immune tolerance resulting in β-cell destruction [15]. In fact, pancreatic β-cells are destroyed as a consequence of an inflammatory process initiated by lymphocytes of the immune system [40]. Although the evidence presented in this systematic review and meta-analysis, suggest that NK cells are activated in patients with T2D, only a few studies reported on effect measures associated with the pathogenesis of T2D. It is well-established that patients with T2D present with dysregulated immune responses and dyslipidaemia, as a consequence of altered lipid metabolism which is associated with an increased risk of developing CVD [41].

The evidence presented in this systematic review and meta-analysis was limited by the unavailability of study-level data of effect measures in some of the included studies, which affected pre-specified quantitative analysis described. Moreover, majority of the included were cross-sectional studies with a high risk of bias, particularly in the internal and external validity domains. Therefore, caution should be taken in the interpretation of these findings and extrapolation of these findings in different populations.

## Conclusion

Overall, the evidence presented in this review suggests that the changes in NK cell counts in patients living with T2D remain unclear and further studies are needed.

## Methods

### General guidelines applied in the current systematic review

This systematic review and meta-analysis was reported according to the Meta-analysis Of Observational Studies in Epidemiology (MOOSE) guidelines [18]. The review protocol was also registered on PROSPERO (CRD42018106159) and has been published [36]. We included randomised control trials, cross-sectional, case-control studies assessing the role of NK cells in adult (≥ 18 years) patients living with T2D.

### Search strategy and study selection

A comprehensive search strategy was developed in consultation with a subject librarian (Table 1S). The search was conducted to identify and retrieve studies reporting on relevant findings that would address the following research questions:

**Research question:** Are NK cells elevated in patients with T2D?

The search strategy comprised of medical subheadings (MeSH) and corresponding keywords which included; type 2 diabetes mellitus, natural killer cells, and cardiovascular disease. The search strategy was used to search the MEDLINE, Cochrane Library and Embase electronic databases using the PUBMED and google scholar search engines. The search was restricted to available full-text human studies published from inception up until 13 November 2019. We also searched the OpenGrey (System for Information on Grey Literature in Europe) (www.opengrey.eu) although these were not included, the bibliographies were screened for relevant citations. In addition, the bibliography of included studies was also screened for additional relevant studies. We used the Mendeley referencing manager (V1.19.10) to manage the included studies and to remove duplicates.

### Primary outcomes

The levels of peripheral blood NK cells were the primary outcome of this systematic review and meta-analysis.

### Risk of bias and quality of the studies

The potential risk of bias in the included study was independently assessed by two reviewers (VM and TMN) using the modified Downs and Black checklist [37] which has four domains namely; reporting bias (10 items), external validity (3 items), internal validity (6 items) and selection bias (7 items). In instances of disagreements, a third reviewer (BBN) was consulted for arbitration. The studies were rated as; excellent if the score was (24–28 points), good if (19–23 points), fair if (13–18 points) and poor if (≤12 points). The levels of inter-rater agreement were assessed using the Cohen’s kappa [37] and the funnel plots were used to assess publication bias.

### Statistical analysis

The meta-analysis of pooled data was performed using Review Manager V.5 (Cochrane Collaboration, Oxford, UK). The level of statistical heterogeneity among the included studies was assessed using Higgin’s *I*^*2*^ index statistic. An *I*^*2*^ value of > 50% was considered as substantial heterogeneity [38]. To explore the sources of heterogeneity within the included studies, a subgroup analysis was performed based on the effect measures reported. Random-effects model was used in the meta-analysis due to substantial levels of heterogeneity amongst the included studies. Moreover, effect estimates were reported as the mean difference (MD) or the standardised mean difference (SMD), and the 95% confidence. The Cohen’s kappa was used to assess the inter-rater reliability and a *p-*value of less than 0.05 was statistically significant.

## Supplementary information

**Additional file 1: Table 1S.** PUBMED search strategy. Search Strategy run on 13 November 2019. **Table 2S.** Sensitivity analysis of studies included in meta-analysis reporting on the levels NK cell in T2D adult patients compared to healthy controls. **Table 3S.** Risk of bias assessment.

## Data Availability

All data generated or analysed during this study are stated in this published article [and its supplementary information files].

## Abbreviations

NK cells: Natural killer cells
SMD: Standardised mean difference
LDL: Low density lipoprotein
MeSH: Medical subject headings
T2D: Type 2 diabetes mellitus
TNF-α: Tumour necrosis factor alpha
CVD: Cardiovascular disease
Th1: T helper cell 1
