# Supplementary material for: Natural killer cell levels in adults living with type 2 diabetes: a systematic review and meta-analysis of clinical studies
Source: BMC Immunol. 2020 Sep 9;21:51. doi: 10.1186/s12865-020-00378-5 (PMC7487809; doi:10.1186/s12865-020-00378-5)
Supplement: Supplementary file 1 — Additional file 1: Table 1S. PUBMED search strategy. Search Strategy run on 13 November 2019. Table 2S. Sensitivity analysis of studies included in meta-analysis reporting on the levels NK cell in T2D adult patients compared to healthy controls. Table 3S. Risk of bias assessment. [file 12865_2020_378_MOESM1_ESM.docx]

**Natural killer cell and cardiovascular-risk profiles in adults living with Type 2 Diabetes Mellitus: A Systematic Review and Meta-Analysis of Clinical Studies**

Vuyolwethu Mxinwa^1^, Phiwayinkosi V. Dludla ^2,3^, Tawanda M. Nyambuya^1,4^, Kabelo Mokgalaboni^1^, Sithandiwe E. Mazibuko-Mbeje^3,5^, Bongani B. Nkambule^1^

^1^School of Laboratory Medicine and Medical Sciences (SLMMS), College of Health Sciences, University of KwaZulu-Natal, Durban, South Africa.

^2^Department of Life and Environmental Sciences, Polytechnic University of Marche, Ancona, Italy.

^3^Biomedical Research and Innovation Platform, Medical Research Council, Tygerberg,

South Africa.

^4^Department of Health Sciences, Faculty of Health and Applied Sciences, Namibia University of Science and Technology, Windhoek 9000, Namibia.

^5^Division of Medical Physiology, Faculty of Health Sciences, Stellenbosch University, Tygerberg 7505, South Africa.

**Corresponding author:**

Bongani B. Nkambule, PhD

School of Laboratory Medicine and Medical Sciences (SLMMS), College of Health Sciences, University of KwaZulu-Natal, Durban, South Africa. Private Bag X54001, Durban, 4000. Email address: [nkambuleb@ukzn.ac.za](mailto:nkambuleb@ukzn.ac.za). Tel: +27-31-260-8964.

| **Concept 1: Natural killer cells** | **Associated words to be searched (MeSH OR textwords)** |
| --- | --- |
| **PUBMED (hits= 30 738)**  "Killer Cells, Natural"[Mesh] | Innate immunity  NK cells |

**Table 1S: PUBMED search strategy**

**Search Strategy run on 13 November 2019**

| **Concept 2: Diabetes Mellitus** | **Associated words to be searched (MeSH OR textwords)** |
| --- | --- |
| **PUBMED (hits= 401 837)**  "diabetes mellitus"[MeSH Terms] OR diabetes mellitus[Text Word] | Glucose intolerance;  Hyperglycaemia  Insulin resistance  Hyperinsulinemia |

COMBINE CONCEPT 1 AND CONCEPT 2

("Diabetes Mellitus, Type 2"[Mesh]) AND "Killer Cells, Natural"[Mesh] = **38 Hits**

**Table 2S.** Sensitivity analysis of studies included in meta-analysis reporting on the levels NK cell in T2D adult patients compared to healthy controls.

| **Outcome : Natural killer cells in the low-risk of bias subgroup** | | | | | |
| --- | --- | --- | --- | --- | --- |
| **Parameter** | **Number of studies** | **Studies omitted** | **MD (95%CI)** | **I^2^ (%), p-value** | **Overall effect:**  **Z, p-value** |
| **Smoking status** | | | | | |
| Smokers | 1[1] | 2 [2, 3] | 0.50 [-0.46, 1.46] | Not applicable | 1.02, p=0.31 |
| Non-smokers | 2 [2, 3] | 1 [1] | 4.81 [4.70, 4.92] | 0%, p<0.0001 | 84.44, p=0.0001 |
|  |  |  |  |  |  |

**Table 3S. Risk of bias assessment**

**References**

1. Olson NC, Doyle MF, de Boer IH, et al (2015) Associations of Circulating Lymphocyte Subpopulations with Type 2 Diabetes: Cross-Sectional Results from the Multi-Ethnic Study of Atherosclerosis (MESA). PLoS One 10(10):1–14. https://doi.org/10.1371/journal.pone.0139962

2. Xiaohong Lv, Gao Y, Dong T, Yang L (2018) Role of Natural Killer T ( NKT ) Cells in Type II Diabetes-Induced Vascular Injuries. 8322–8332. https://doi.org/10.12659/MSM.912446

3. Piatkiewicz P (2013) The Dysfunction of NK Cells in Patients with Type 2 Diabetes and Colon Cancer. 245–253. https://doi.org/10.1007/s00005-013-0222-5

4. Åkesson C, Uvebrant K, Oderup C, et al (2010) Altered natural killer (NK) cell frequency and phenotype in latent autoimmune diabetes in adults (LADA) prior to insulin deficiency. Clin Exp Immunol 161(1):48–56. https://doi.org/10.1111/j.1365-2249.2010.04114.x

5. Simar D, Versteyhe S, Donkin I, et al (2014) DNA methylation is altered in B and NK lymphocytes in obese and type 2 diabetic human. Metabolism 63(9):1188–1197. https://doi.org/10.1016/j.metabol.2014.05.014

6. Lynch LA, O’Connell JM, Kwasnik AK, Cawood TJ, O’Farrelly C, O’Shea DB (2009) Are natural killer cells protecting the metabolically healthy obese patient? Obesity 17(3):601–605. https://doi.org/10.1038/oby.2008.565

7. Berrou J, Fougeray S, Venot M, Chardiny V (2013) Natural Killer Cell Function , an Important Target for Infection and Tumor Protection , Is Impaired in Type 2 Diabetes. 8(4). https://doi.org/10.1371/journal.pone.0062418

8. Medellín-Garibay SE, Cortez-Espinosa N, Milán-Segovia RC, et al (2015) Clinical pharmacokinetics of rifampin in patients with tuberculosis and type 2 diabetes mellitus: Association with biochemical and immunological parameters. Antimicrob Agents Chemother 59(12):7707–7714. https://doi.org/10.1128/AAC.01067-15

9. Nekoua M, Fachinan R, Atchamou AK, et al (2016) Modulation of immune cells and Th1/Th2 cytokines in insulin-treated type 2 diabetes mellitus. Afri Heal Sci 16(3):712–724. https://doi.org/10.4314/ahs.v16i3.11

10. Nam HW, Cho YJ, Lim JA (2018) Functional status of immune cells in patients with long-lasting type 2 diabetes mellitus. 125–136. https://doi.org/10.1111/cei.13187

11. Singh K (2019) Cellular immunological changes in patients with LADA are a mixture of those seen in patients with type 1 and type 2 diabetes. 64–73. https://doi.org/10.1111/cei.13289

12. Guo H, Xu B, Gao L, Sun X, Qu X, Li X (2013) High frequency of activated nat- ural killer and natural killer T-cells in patients with new onset of type 2 diabetes mellitus. Experimental Biology and Medicine. https://doi.org/10.1258/ebm.2012.011272

13. Kim JH, Park K, Lee SB, Kang S, Park JS (2019) Relationship between natural killer cell activity and glucose control in patients with type 2 diabetes and prediabetes. 10(5):1223–1228. https://doi.org/10.1111/jdi.13002
